# Inflammatory role of toll‐like receptors in the hippocampus of 3xTg‐AD animals

**DOI:** 10.1002/alz.089907

**Published:** 2025-01-09

**Authors:** Carlos Wagner Leal Cordeiro Júnior, Vanessa V.J. De‐Paula, Caíque de Oliveira, Portugal Couto

**Affiliations:** ^1^ University of Sao Paulo (USP), São Paulo, São Paulo Brazil; ^2^ Laboratory of Neurosciences (LIM23), Institute of Psychiatry, Faculty of Medicine, University of São Paulo, São Paulo Brazil; ^3^ USP, São Paulo Brazil; ^4^ Universidade de São Paulo (USP), São Paulo Brazil

## Abstract

**Background:**

Alzheimer's disease (AD) is a progressive neurodegenerative disease with multifactorial etiology. The toxicity of senile plaques and neurofibrillary tangles is associated with changes in the clearance of tau and beta‐amyloid proteins, which are closely related to inflammatory imbalances. Pro‐inflammatory cytokines are present in abnormal details due to the overactivation of immunological pathways, especially toll‐like receptors. Our objective is to determine the difference between the proteins altered in AD and the inflammatory role of toll‐like receptors in the hippocampus of 3xTg‐AD animals chronically treated with lithium at therapeutic and subtherapeutic doses and how lithium acts in this regulation.

**Method:**

48 mice (3xTg‐AD;Tg) were treated with lithium for 3 months and divided into 3 groups (control, 1mM Li and 2mM Li). Hippocampal tissue was subjected to proteome. The inflammatory pathways of the identified proteins were compared by the STRING software and the molecular functions were analyzed by Gene Ontology, using the ClueGO software. The Toll‐Likes receptor family genes were analyzed together with Tau and amyloid beta proteins in the STRING bioinformatics program, in order to study the ontological pathways and molecular function. As parameters, we set significance at 0.7 with a limitation of 50 interactions per gene. The project was submitted to the Ethics Committee under protocol (1876/2022).

**Result:**

The genes of the Toll‐Like receptor family were analyzed using the STRING bioinformatics program to study ontological pathways and molecular function. As parameters, we set significance at 0.7 with a limitation of 50 interactions per gene. Thus, the results demonstrate that ontological changes include: signal transduction activating the immune response, activation of the immune response, regulation of defense response, positive regulation of the immune response, regulation of the stress response, immune response, positive regulation of the response to stimulus, and the process of the immune system.

**Conclusion:**

The results appear promising for understanding the inflammatory pathways related to toll‐like receptors. Since the identification of positively regulated immune responses suggests that lithium, considering its modulating effect on both proteins involved in neurodegeneration and associated inflammatory processes. However, we emphasize the need for additional clinical studies to validate and extend these findings in animal models.

